# Man vs machine in emergency medicine – a study on the effects of manual and automatic vital sign documentation on data quality and perceived workload, using observational paired sample data and questionnaires

**DOI:** 10.1186/s12873-018-0205-2

**Published:** 2018-12-13

**Authors:** Niclas Skyttberg, Rong Chen, Sabine Koch

**Affiliations:** Department of Learning, Informatics, Management and Ethics, Health Informatics Centre, 171 77 Stockholm, Sweden

**Keywords:** Data quality, Automated documentation, Vital signs, Emergency department, Emergency medicine

## Abstract

**Background:**

Emergency medicine is characterized by a high patient flow where timely decisions are essential. Clinical decision support systems have the potential to assist in such decisions but will be dependent on the data quality in electronic health records which often is inadequate. This study explores the effect of automated documentation of vital signs on data quality and workload.

**Methods:**

An observational study of 200 vital sign measurements was performed to evaluate the effects of manual vs automatic documentation on data quality. Data collection using questionnaires was performed to compare the workload on wards using manual or automatic documentation.

**Results:**

In the automated documentation time to documentation was reduced by 6.1 min (0.6 min vs 7.7 min, *p* <  0.05) and completeness increased (98% vs 95%, *p* <  0.05). Regarding workflow temporal demands were lower in the automatic documentation workflow compared to the manual group (50 vs 23, *p* <  0.05). The same was true for frustration level (64 vs 33, *p* <  0.05). The experienced reduction in temporal demands was in line with the anticipated, whereas the experienced reduction in frustration was lower than the anticipated (27 vs 54, *p* < 0.05).

**Discussion:**

The study shows that automatic documentation will improve the currency and the completeness of vital sign data in the Electronic Health Record while reducing workload regarding temporal demands and experienced frustration. The study also shows that these findings are in line with staff anticipations but indicates that the anticipations on the reduction of frustration may be exaggerated among the staff. The open-ended answers indicate that frustration focus will change from double documentation of vital signs to technical aspects of the automatic documentation system.

**Electronic supplementary material:**

The online version of this article (10.1186/s12873-018-0205-2) contains supplementary material, which is available to authorized users.

## Background

Emergency medicine is dedicated to the diagnosis and treatment of unforeseen illness or injury. Many practitioners specialized in emergency medicine work in emergency departments or other settings where patient flow usually is high and the outcome of decisions is time-dependent. In this setting, the use of clinical decision support systems (CDSS) could have a potential to transform workflow and improve clinical outcome thereby reducing workload and improving quality. However, to release the potential of CDSS there are many issues to solve [1–3]. One challenge in emergency medicine is that information needed for decision support may not be present in the electronic health records (EHRs). Also, even if the information is present it may not be of adequate quality for the CDSS to work properly [4]. There is growing evidence to show that the data quality in the emergency departments’ electronic health records is inadequate for reuse by CDSS [5].

In emergency medicine, vital signs are of great importance for many decisions. Data quality of the vital signs has been questioned and improvements suggested [6, 7]. Manual documentation of the vital signs may be linked to both low completeness and a delay before the data is available for decision support. One proposed improvement is to increase the automation of vital sign measurement and documentation and thereby reducing time to documentation and increase completeness. Increased automation also has the potential to reduce workload for the staff thereby improving situational awareness and team performance [8].

Automatic data capture for vital signs has been studied in anesthesia and intensive care and has been shown to increase the completeness of data and currency of the recordings, effects of correctness have been uncertain [9]. In intensive care and anesthesia, patients are stationary and continuously connected to measurement devices. Less is known about automatic data capture in the emergency department setting, where there is a high flow of patients and where staff, patients and measurement equipment are mobile.

### Objective

The aim of this study was to compare the effects of manual and automatic documentation of vital sign measurements on data quality and perceived workload in emergency medicine, a context with high patient turnover and a mobile workflow.

## Methods

This study used the STROBE reporting guidelines [10].

### Study setup

We performed an observational study of 50 patients and 200 vital sign measurements at an emergency department (ED) in one hospital in Stockholm, Sweden. The ED at the study hospital handles around 90,000 patient visits a year and is one of the three largest EDs in Sweden. To study effects on workload by automation of vital sign documentation a questionnaire study was designed to collect data for comparison of the perceived workload according to the NASA-TLX workload assessment [11–13]. The questionnaire data collection was performed at two separate emergency wards at the hospital with different workflows regarding vital sign documentation.

### Inclusion and exclusion criteria for the data quality assessment

Patients were recruited at the triage in the ED, the observer included all arriving patients at their triage unless already involved in an ongoing observation. As soon as an observation was completed the next triaged patients were given the opportunity to participate. Both walk-in patients and patients arriving by ambulance were included. Participation was voluntary, participants had to opt-in after written information was provided and the only exclusion criteria was the inability to consent to the trial. All data were anonymized after collection.

### Data collection of vital sign measurements

**Fig. 1:**
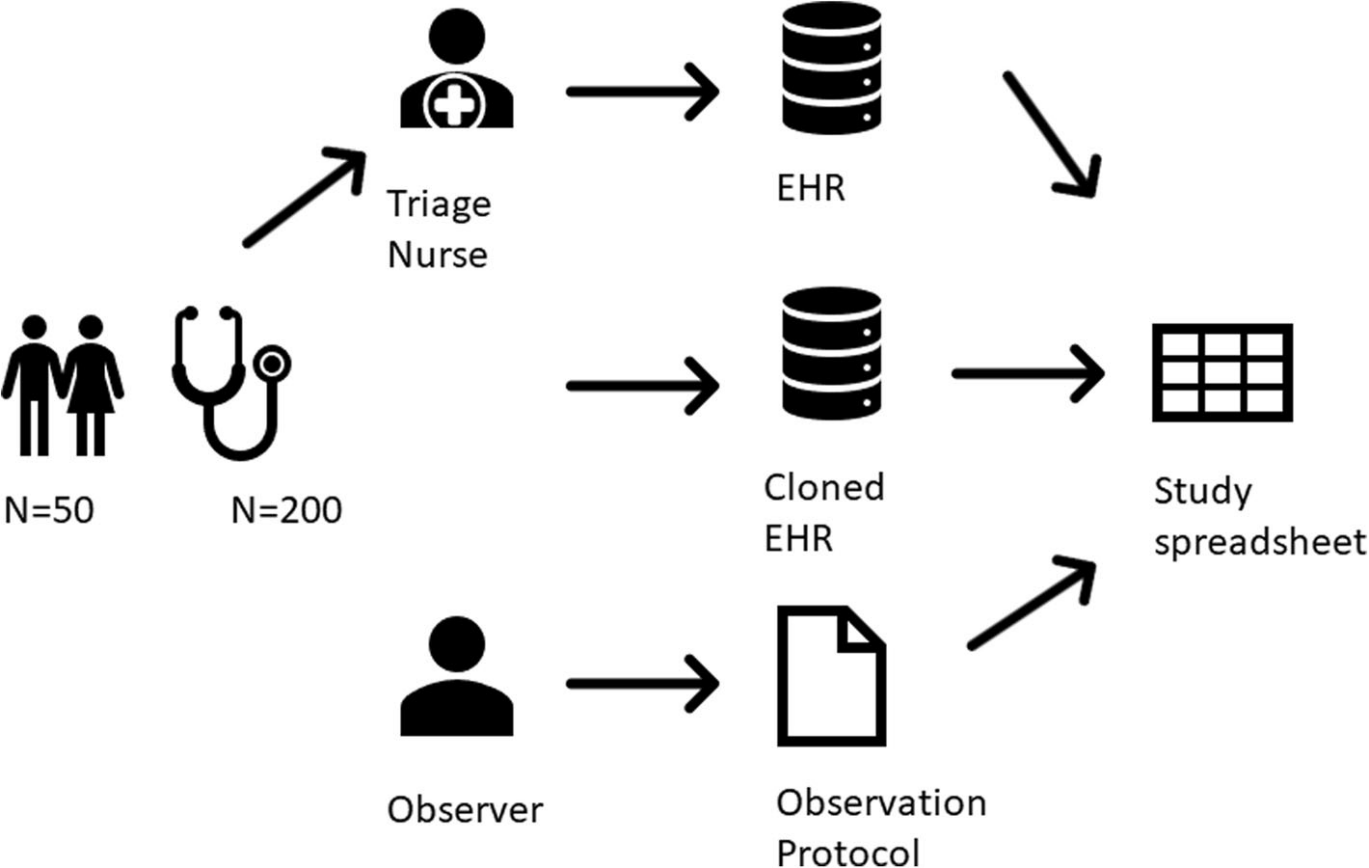
Data collection during observations. A trained observer observed the triage and documented according to the study protocol, while the triage nurse followed normal documenting procedures in the EHR, the measurement equipment transferred the measurements to the cloned EHR database. All data was collected in an anonymized spread sheet

In the ED, we compared standard manual documentation practice to an automated documentation workflow. In both workflows, the vital signs (systolic blood pressure, tympanic temperature, oxygen saturation, and heart rate) were measured. In the manual workflow the vital signs were documented in the Electronic Health Record (EHR) System according to the standard manual practice and in the automated workflow, the measurements were automatically transferred from the measurement device to a copied version of the EHR (Fig. 1).

The measurement and the documentation processes were observed by a clinically trained observer and data was collected with a structured protocol including the patient identifier, arrival time, observed vital sign measurement time, observed results of vital sign measurements and the area of chief complaint structured as orthopedic, surgical or internal medicine. From the two studied workflows data regarding time of documentation and documented results from the vital sign measurements, were extracted from the receiving systems (EHR and EHR copy) and added to the observation protocol.

### Recruitment of participants and data collection for assessment of workload

Two emergency wards were studied for evaluation of perceived workload, one which had implemented automatic documentation and one which was still using the hospital’s standard manual workflow. Both wards were focusing on patients with unplanned emergency visit. The ward using a manual documentation workflow had a focus on general medicine and the ward with an automated documentation workflow focused on orthopedic emergencies. Both wards were working according to hospital routines based on the New Early Warning Score guidelines. Both wards used the same type of mobile measurement device (Welsh-Allyn, Skaneateles Falls, USA) and the same EHR system (Cambio COSMIC, Stockholm, Sweden) for documentation. To evaluate workload a questionnaire was developed and evaluated in a pilot study. In the questionnaire data collection, an e-mail invite was sent out using the hospital mail addresses to nurses at the studied wards, the email contained information about the study, that participation was voluntary and that all data was anonymized. The questionnaires were digital and participants had to opt-in by using a web-link to the questionnaire. Two reminders were sent out to all nurses at the wards, but no other follow up was done. A total of 246 questionnaires were sent out and 70 completed questionnaires were received, corresponding to a 28% response rate. The low response rate can to some extent be explained by that the e-mail groups that were used for sending the questionnaires are updated with new employees, but old employees are not always removed and those that are on parental leave or those only working occasionally at the wards would not be expected to answer. After discussion with the managers at the wards and with the HR unit at the hospital an expected number of employees was set to 200, making the adjusted response rate to about 50%.

The questionnaire was developed from the NASA-TLX workload assessment [11], which has been used in the assessment of workload effects of information technology and system support in health care [13, 14]. The NASA-TLX questionnaire focuses on six aspects of workload: mental demand, physical demand, temporal demand, effort, performance, and frustration. The questionnaires used the NASA-TLX questions to assess the present situation for the nurses at the departments. For the nurses using manual documentation, the questions were also used to assess what effects they would expect upon a switch from manual to automated documentation. The nurses using automatic documentation got a questionnaire focusing on the present situation and the experienced effects of the switch from manual to automatic documentation. The questionnaire also contained background information on the profession, years employed at the hospital, years of experience in the profession, gender and to what extent the participant measured and documented vital signs in everyday practice. All questionnaires contained an open-ended free text field where the participants were encouraged to give their opinion on the current workflow and give an opinion about automation in the documentation of measured vital signs. The questionnaires are provided in Additional file 1: Appendix 1.

### Data analysis

For each observed measurement completeness, correctness and currency were evaluated for the documentation in the EHR and in the EHR copy. Each observed measurement was connected both to documentation in the EHR and in the EHR copy so that a set of paired samples was created. All data analysis was done using SPSS Statistics (IBM, 2012).

### Currency

We calculated “time to documentation” defined as the time from the observed measurement to the time stamp of entry in the receiving system, for the manual workflow the time stamp was extracted from the EHR and for the automatic group the time stamp was extracted from the EHR copy. A mean time to documentation was calculated for the standard and the automatic documentation workflows and the difference was evaluated with confidence intervals and calculation of *p*-values using the student’s t-test.

### Correctness

To assess correctness an acceptable deviation from the observed value was defined (Table 1). The acceptable deviation was defined from a clinical perspective, discussed and decided upon by the research team, a senior intensive care consultant and a senior emergency care consultant. For each observation of a vital sign measurement, the documentation in the EHR and the cloned EHR was evaluated and graded as correct or incorrect, this way a set of paired nominal data samples was created and MacNemars test used to evaluate homogeneity between the samples. A difference between the observed value and the documented value was also calculated for each measurement and the mean difference for each vital sign was evaluated by calculation of confidence intervals and *p* values using the students’ t-test.

**Table 1:** Acceptable deviation from observed values

Vital Sign	Acceptable deviation from observation
Systolic Blood pressure	<= 4 mm/Hg
Tympanic Temperature	<=0.1 degree Celsius
Saturation	< 2%
Heart Rate	< = 4 beats per minute

### Completeness

Completeness was calculated in percent of vital sign measurements present for each documentation method. For each observed measurement of a vital sign, the corresponding entry in the EHR and EHR copy was evaluated and deemed either complete or incomplete, generating a set of nominal paired data on completeness. McNemar’s test for paired data was used to evaluate significance for completeness and correctness.

### Workload

All data from the questionnaires were entered into a spreadsheet (Microsoft Excel) and further analysis was done using data analysis was done using SPSS Statistics (IBM, 2012).

For perceived workload, all scales were rated from very low (0) to very high (100). An average value was calculated for each group and the student’s t-test was used to evaluate the statistical significance of the variation between the means.

For the difference between the expected vs experienced change of workload with a shift to automated documentation a scale from reduced (100) to increased (− 100) workload was used. The numbers were not visible to the participants as only a visual scale was used. A mean change was calculated for each category in the two groups and statistical significance was evaluated with the student’s t-test.

## Results

### Currency

**Fig. 2:**
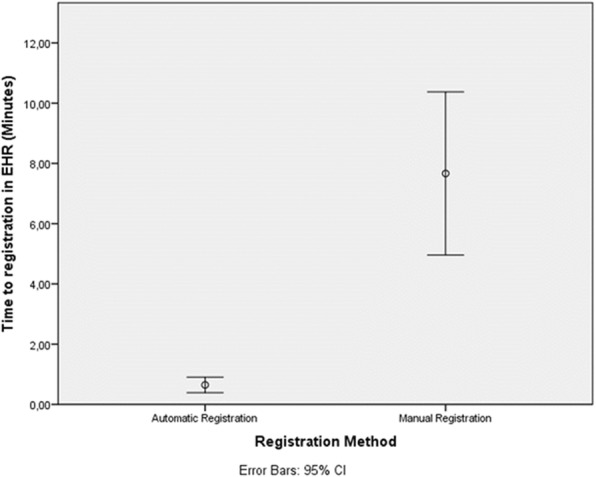
Time to registration for manual and automatic documentation. Mean time to documentation in the EHR for the manual and automatic documentation with error bars showing the 95% Confidence Interval

The mean time to documentation was 0.6 min (CI 0,4-0,9) in the automatic group and 7.7 (CI 5.0–10) minutes in the manual documentation workflow, a difference of 6.1 min between the means (*P* < 0.01) (Fig. 2).

### Correctness

In the automatic documentation workflow, 98% of registrations were correct, according to the defined acceptable deviation (Table 1), when compared to the observed data. In the manual documentation workflow, 95% of the registrations were found to be correct. McNemar’s test did not show any significant difference (*p* = 0.61). When evaluating the absolute deviations from the observed values no statistically significant differences were found between the automatic and manual documentation workflow (Table 2).

**Table 2:** Deviations from observed values in the manual and automatic workflow

		Manual workflow	Automatic	*p*-value
		Mean deviation from observation (CI 95%)	Mean deviation from observation (CI 95%)	
Systolic Blood Pressure	mm/Hg	−4.1 (−9.9–1.7)	0.22 (−0.2–0.6)	ns
Temperature	Degree Celsius	2.95 (−0.2–6.2)	0.03 (0–0.1)	ns
Saturation	%	1.58 (0–3.2)	1.62 (−0.8–4.0)	ns
Heart Rate	Beats Per Minute	8.9 (2.9–15)	4.2 (2–6.4)	ns

### Completeness

In the automatic documentation workflow, all 196 out of the 200 observed measurements had corresponding registrations present in the test environment making completeness 98%. In the manual documentation workflow, a 190 of the 200 measurements were documented in the EHR making completeness 95%. McNemar’s test showed a significant difference in the level of completeness between the documentation methods (*p* < 0.05).

### Background data of the questionnaire

The ward with normal documentation is a larger ward with more employed staff as shown by the number of questionnaires sent (149 vs 97). The response rate was similar in both groups although the degree of completed questionnaires was somewhat lower in the automatic group (Table 3). Subgroup analysis showed that the group who answered that they “almost never” worked with vital signs had a low completion rate in the questionnaire.

**Table 3:** Background data of the questionnaire

	Manual Documentation	Automatic Documentation
Questionnaires sent	149	97
Replies (n)	53	33
(n) Complete answers	50	24
Reply frequency	36%	34%
Gender Female%	85%	88%
Registered Nurses%	72%	52%
Nurse assistants%	28%	48%
> 5 years in profession %	77%	70%
> 5 years at the hospital %	51%	65%
How often do you measure vital signs in your work?	(*n* = 53)	(*n* = 33)
Almost every day	83%	67%
Less than every other day	15%	
Almost never	2%	33%

### Perceived workload

When comparing the means in the manual and automated documentation group (Table 4) it shows that the Temporal demand, 50 (47–53 CI95%) vs 23 (14–31 CI95%), and the Frustration level 63 (59–66 CI95%) vs 33 (22–45 CI95%) are rated significantly lower in the automated documentation workflow. This shows that staff feel less frustrated and perceive that they spend less time on vital sign measurement and documentation in an automated documentation workflow. Both groups had high ratings in performance indicating they were content with their level of performance regardless of documentation workflow. The lowest ratings were in the physical demand category.

**Table 4:** Workload in the manual and automatic documentation workflow according to the NASA- TLX questionnaire

	Manual Documentation Mean (CI 95%) (*n* = 50)	Automated Documentation Mean (CI 95%) (*n* = 24)	*p*
Mental demand. How much mental activity is required for the measurement and documentation of vital signs?	33 (31–35)	34 (24–43)	ns
Physical demand. How much physical activity is required in the measurement and documentation of vital signs?	23 (21–26)	16 (10–21)	ns
Temporal demand. How much time pressure do you experience due to the demand of vital sign measurement and documentation?	50 (47–53)	23 (14–31)	< 0.05*
Performance. How satisfied are you with your performance at measuring and documenting the vital signs?	68 (66–69)	73 (65–81)	ns
Frustration level. How much frustration do you experience with regards to the tasks of measuring and documenting the vital signs?	63 (59–66)	33 (22–45)	< 0.05*
Effort. How hard do you have to work (mentally and physically) to accomplish your level of performance?	44 (42–45)	36 (31–40)	ns

### Anticipated vs experienced change in workload with automated documentation

Only the frustration category showed significant differences between the anticipated and experienced change in workload when comparing automated and manual documentation (Table 5). The study shows that there may be overinflated expectations in the reduction of frustration because the anticipation of reduced frustrations was rated higher 54 (44–64 CI95%) than the experienced reduction in frustration 27 (10–43 CI95%). This is a somewhat contrasting finding to the first part of the questionnaire where frustration is rated significantly lower in the automated group. The anticipation of a reduction of temporal demand was given the highest rating in both groups 57 (47–66 CI95%) vs 47 (32–66 CI95%) with no statistically significant difference between the means. This indicates that there is a large anticipation of a reduction in time spent in documentation and measurement of vital signs, this anticipation seems well founded as it is also highly rated in the experienced outcome. This gives support to the finding in the first part of the questionnaire where temporal demands are significantly lower in the automated group.

**Table 5:** Expected and experienced effects on workload by automating vital sign documentation

	Expected (CI 95%)	Experienced (CI 95%)	*p*
Mental demand. How do you expect a switch to automated documentation will change mental demand vs How did you experience that the switch to automated documentation changed mental demand?	56 (46–66)	40 (23–56)	ns
Physical demand. How do you expect a switch to automated documentation will change physical demand vs How did you experience that the switch to automated documentation changed physical demand?	24 (15–33)	41 (26–56)	ns
Temporal demand. How do you expect a switch to automated documentation will change temporal demand vs How did you experience that the switch to automated documentation changed temporal demand?	57 (47–66)	47 (32–66)	ns
Performance. How do you expect a switch to automated documentation will change your performance vs How did you experience that the switch to automated documentation changed your performance?	52 (40–63)	39 (21–56)	ns
Frustration level. How do you expect a switch to automated documentation will change your frustration vs How did you experience that the switch to automated documentation changed your frustration?	54 (44–65)	27 (10–43)	< 0.5
Effort. How do you expect a switch to automated documentation will change your effort vs How did you experience that the switch to automated documentation changed your effort?	45 (38–53)	38 (26–51)	ns

### Open-ended comments in the questionnaire

One of the comments supporting the currency and temporal gains of a switch from a manual to an automatic workflow stated “*It takes me at least a minute to document the vital signs in the EHR, given that I am not interrupted, and some days we triage 30-40 patients a shift. I am convinced we spend more than 30 minutes a shift just documenting the vital signs. It feels unnecessary.*”. In the manual workflow the nurse assistants could make the measurements but not document them in the EHR, instead, they documented on a paper template and gave it to the nurse who entered the values into the EHR. This gave rise to questions “*As a nurse assistant I am not allowed to document in the EHR. If we switch to an automated documentation will I still be allowed to make the measurements?*”. Further concerns in the manual workflow included what values were transferred to the EHR “*There may be errors in the measurements, saturation may be low in a cold patient, so there has to be some manual check before the measurements are automatically documented.*”. In the automated documentation, group satisfaction seemed generally high but there were concerns and frustration with the technical stability of the automatic documentation “*Overall it is a very positive experience with automatic transfer, although it is frustrating when there are technical problems. In those cases, sometimes we make double measurements and get double documentation in the EHR. Sometimes the values are lost. We tend to write the measurements on notes too, just for safety.*” However, not all comments signalled insecurity with the new system “*I feel confident that the right values get documented in the right patient chart*”.

## Discussion

The main contribution of this work is that it shows that switching to an automated documentation practice can be done in a mobile emergency department where patient turnover is high, with increased data quality and reduced staff workload as an outcome. These findings support earlier work, like Wong et al. who showed that increased digitalization in the documentation at hospital wards may increase data quality and reduce workload [15].

Data quality has been described in three different categories; correctness, currency, and completeness [16]. The factors affecting data quality have been described in earlier studies suggesting that both factors relating to the care process and to information technology are important. These studies show that a lack of standardization and staff training may affect data quality negatively as will a lack of digital support [6, 17]. One of the aims of this study was to quantitatively describe the effects on data quality by automation of vital sign documentation and the results show that switching to an automated documentation workflow will improve completeness and currency but no statistically significant effect on correctness was found. These findings are in line with our earlier retrospective study on vital sign data quality in the emergency department, which shows that manual documentation of vital signs will cause delays in registration and lower completeness [4, 6]. Studies in anesthesia and intensive care have shown similar outcomes [9] and the effect on correctness has been questioned partly because a too high degree of automation may lead to capture and documentation of incorrect data, which is described in one of the quotes in the questionnaire: “*There may be errors in the measurements, saturation may be low in a cold patient, so there has to be some manual check before the measurements are automatically documented*”.

The gain in currency is important in emergency medicine because the time to diagnosis and treatment is strongly connected to the outcome for the patients. If warning scores for severe illness are to be calculated it is important that they are provided at the right time to the team treating the patients. Earlier work has shown that the data quality in emergency medicine may be inadequate to introduce clinical decision support systems [5, 18] and one main issue may be the low currency of the data. In this study, the gain in time to documentation was 6.1 min, but it also worth noting the higher variability shown in the confidence intervals for the two different workflows. This indicates that the manual documentation practice is a workflow with much higher variability and in a standardized process, high variability is generally regarded as a quality problem.

Automation of tasks should be connected to a reduced workload for the staff [8]. This study shows that the workload associated with measurement and documentation of vital signs is lower in an automated documentation workflow. Gains are according to our findings significant in the temporal demands and the frustration categories. The reduced temporal demand is both anticipated and experienced by the staff in this study and the combined findings in this study show that investing in automation of vital sign documentation will save time for the staff and improve the currency of the vital sign data. Introducing automation in emergency medicine may lead to task shifting and changes in the workflow. One of the comments in the manual group showed that assisting nurses, although performing the measurement were not allowed to document the result in the EHR. In the automated group assisting nurses did perform the measurement and the device sent the vital signs directly to the EHR. This change of the workflow likely reduced temporal demands by taking out a handover step in the process.

The frustration level was lower in the automated documentation flow, but it seems like the staff was expecting more according to the anticipated vs experienced outcome. Looking at the comments it seems like frustration focus has changed from the manual documentation which is perceived as unnecessary “*I am convinced we spend more than 30 minutes a shift just documenting the vital signs. It feels unnecessary.”* to technical issues with the automatic documentation “*is frustrating when there are technical problems”.*

The gain in data quality and reduced workload may be of importance for quality and patient safety as other studies have shown that reduced workload among the staff is associated with increased situation awareness [19]. The increased data quality may also be a prerequisite for further automation and introduction of CDSS in emergency medicine. Further automation may lead to increased team performance and thereby contribute to additional effects on quality and safety in health care.

### Limitations

The study population is small and there was a low response rate in the questionnaire’s data collection. The small number of patients included are ameliorated by the fact that a multiple of measurements was done for each patient. Still, the study may have been underpowered to show differences regarding correctness in the vital signs. The findings could be transferrable to contexts where vital signs are manually measured and documented in the EHR.

### Further studies

This study does not aim to follow up direct effects regarding patient safety and quality of care. This is an important subject to cover because concerns have been raised that increased automation may delay clinical response to deteriorating patients if the clinicians do not manually see and consider the vital signs. The authors recommend and plan further studies in the field of automation effects on outcomes in clinical care.

## Conclusion

This study shows that automated documentation of vital signs will increase the currency and completeness of vital sign data while reducing the temporal demands and frustration level associated with measurement and documentation of vital signs among the staff. These findings are important when discussing the outcomes of automation in emergency medicine and give direction to decisions on investments in information technology.

## Additional file

Additional file 1: Appendix 1 – Questionnaires used in workload assessment. (DOCX 20 kb)

## Abbreviations

CDSS: Clinical Decision Support Systems
ED: Emergency Department
EHR: Electronic Health Record

## References

[1] Bennett P, Hardiker NR (2016). The use of computerized clinical decision support systems in emergency care: a substantive review of the literature. J Am Med Informatics Assoc.

[2] Musen MA, Middleton B, Greenes RA (2014). Clinical decision-support systems. Biomedical informatics.

[3] Feldman SS, Buchalter S, Hayes LW (2018). Health information Technology in Healthcare Quality and Patient Safety: literature review. JMIR Med Informatics.

[4] Skyttberg N, Chen R, Blomqvist H, Koch S (2017). Exploring vital sign data quality in electronic health records with focus on emergency care warning scores. Appl Clin Inform.

[5] Perry WM, Hossain R, Taylor RA (2018). Assessment of the feasibility of automated, real-time clinical decision support in the emergency department using electronic health record data. BMC Emerg Med.

[6] Skyttberg N, Vicente J, Chen R, Blomqvist H, Koch S (2016). How to improve vital sign data quality for use in clinical decision support systems? A qualitative study in nine Swedish emergency departments. BMC Med Inform Decis Mak.

[7] di Martino P, Leoli F, Cinotti F, Virga A, Gatta L, Kleefield S (2011). Improving vital sign documentation at triage: an emergency department quality improvement project. J Patient Saf.

[8] Parasuraman R, Riley V (1997). Humans and automation: use, misuse, disuse, abuse. Hum Factors J Hum Factors Ergon Soc.

[9] Kadry B, Feaster WW, Macario A, Ehrenfeld JM. Anesthesia information management systems: past, present, and future of anesthesia records. Mt Sinai J Med. 79:154–65. 10.1002/msj.21281. 10.1002/msj.21281 22238048

[10] von Elm E, Altman DG, Egger M, Pocock SJ, Gøtzsche PC, Vandenbroucke JP (2007). Strengthening the reporting of observational studies in epidemiology (STROBE) statement: guidelines for reporting observational studies. BMJ.

[11] Hart SG, Staveland LE, Jose S, California SJ (1988). Development of NASA-TLX (task load index): results of empirical and results of empirical and theoretical research. Adv Psychol.

[12] Hart SG (2006). NASA-task load index (NASA-TLX); 20 years later. Proc Hum Factors Ergon Soc Annu Meet.

[13] Hoonakker P, Carayon P, Gurses A, Brown R, Mcguire K, Khunlertkit A (2011). Measuring workload of ICU nurses with a questionnaire survey: the NASA task load index (TLX). IIE Trans Healthc Syst Eng.

[14] Ruiz-Rabelo JF, Navarro-Rodriguez E, Di-Stasi LL, Diaz-Jimenez N, Cabrera-Bermon J, Diaz-Iglesias C (2015). Validation of the NASA-TLX score in ongoing assessment of mental workload during a laparoscopic learning curve in bariatric surgery. Obes Surg.

[15] Wong D, Bonnici T, Knight J, Gerry S, Turton J, Watkinson P. A ward-based time study of paper and electronic documentation for recording vital sign observations. JAMIA. 2017;(4):717–21. 10.1093/jamia/ocw186. 10.1093/jamia/ocw186 PMC7651906 28339626

[16] Weiskopf NG, Weng C (2013). Methods and dimensions of electronic health record data quality assessment: enabling reuse for clinical research. J Am Med Inform Assoc.

[17] Stevenson JE (2018). Factors influencing the quality of vital sign data in electronic health records: a qualitative study. J Clin Nurs.

[18] Mashoufi M, Ayatollahi H, Khorasani-Zavareh D (2018). A review of data quality assessment in emergency medical services. Open Med Inform J.

[19] Endsley MR (1999). Level of automation effects on performance, situation awareness and workload in a dynamic control task. Ergonomics.

