# Supplementary material for: Man vs machine in emergency medicine – a study on the effects of manual and automatic vital sign documentation on data quality and perceived workload, using observational paired sample data and questionnaires
Source: BMC Emerg Med. 2018 Dec 13;18:54. doi: 10.1186/s12873-018-0205-2 (PMC6293611; doi:10.1186/s12873-018-0205-2)
Supplement: Supplementary file 1 — Appendix 1 – Questionnaires used in workload assessment. (DOCX 20 kb) [file 12873_2018_205_MOESM1_ESM.docx]

# Appendix 1 – Questionnaires:

These are the questions in the questionnaires. The questionnaires were digital and sent out through SurveyMonkey ®. These are the translated questions displayed in a similar but not identical way as the digital questionnaires.

# Questionnaire 1 – Used in sites with manual documentation

## Background

| What is your profession | Nurse | Nurse Assistant | Doctor | Other |  |
| --- | --- | --- | --- | --- | --- |
| How long have you been in the profession | < 1 year | 1-2 years | 2-3 years | 4-5 years | > 5 years |
| How long have you worked at the hospital | < 1 year | 1-2 years | 2-3 years | 4-5 years | > 5 years |
| What is your gender identity | Female | Male | Not answering |  |  |
| How often does your work include measurement of vital signs | Most days | Less often than every other day | Almost never |  |  |
| How often does your work include documentation of vital signs | Most days | Less often than every other day | Almost never |  |  |

## Present Workload

| How mentally demanding is the task of measuring and documenting the vital signs | Very low | ________________________________________ | Very high |
| --- | --- | --- | --- |
| How Physically Demanding is the is the task of measuring and documenting the vital signs | Very low | ________________________________________ | Very high |
| How hurried or rushed is the task of measuring and documenting the vital signs | Very low | ________________________________________ | Very high |
| How well do you preform the task of measuring and documenting the vital signs | Very low | ________________________________________ | Very high |
| How much frustration do you experience when performing the task of measuring and documenting the vital signs | Very low | ________________________________________ | Very high |
| Overall, how hard do you have to work when performing the task of measuring and documenting the vital signs | Not hard | ________________________________________ | Very hard |

## Open ended question

| Any other Thoughs you want to share on performing the task of measuring and documenting the vital signs? |  |
| --- | --- |

## Anticipation of automation effects

| Regarding mental demand I believe automation of documentation will | Decrease demand | ________________________________________ | Increase Demand |
| --- | --- | --- | --- |
| Regarding Physical demand - I believe automation of documentation will | Decrease demand | ________________________________________ | Increase Demand |
| Regarding temporal demands - I believe automation of documentation will | Decrease demand | ________________________________________ | Increase Demand |
| Regarding outcome of documentation I believe automation will | Decrease demand | ________________________________________ | Increase Demand |
| Regarding Frustration - I believe automation of documentation will | Decrease Frustration | ________________________________________ | Increase Fustration |
| Overall, I believe automation of documentation will | Decrease demand | ________________________________________ | Increase Demand |

## Open ended question

| Any other thought yo want to share on automatic transfer of vital signs from the measurement device to the EHR? |  |
| --- | --- |

# Questionnaire 2 – Used at sites with automatic documentation of workload

## Background

| What is your profession | Nurse | Nurse Assistant | Doctor | Other |  |
| --- | --- | --- | --- | --- | --- |
| How long have you been in the profession | < 1 year | 1-2 years | 2-3 years | 4-5 years | > 5 years |
| How long have you worked at the hospital | < 1 year | 1-2 years | 2-3 years | 4-5 years | > 5 years |
| What is your gender identity | Female | Male | Not answering |  |  |
| How often does your work include measurement of vital signs | Most days | Less often than every other day | Almost never |  |  |
| How often does your work include documentation of vital signs | Most days | Less often than every other day | Almost never |  |  |

## Present Workload

| How mentally demanding is the task of measuring and documenting the vital signs | Very low | ________________________________________ | Very high |
| --- | --- | --- | --- |
| How Physically Demanding is the is the task of measuring and documenting the vital signs | Very low | ________________________________________ | Very high |
| How hurried or rushed is the task of measuring and documenting the vital signs | Very low | ________________________________________ | Very high |
| How well do you preform the task of measuring and documenting the vital signs | Very low | ________________________________________ | Very high |
| How much frustration do you experience when performing the task of measuring and documenting the vital signs | Very low | ________________________________________ | Very high |
| Overall, how hard do you have to work when performing the task of measuring and documenting the vital signs | Not hard | ________________________________________ | Very hard |

## Open ended question

| Any other Thoughts you want to share on performing the task of measuring and documenting the vital signs? |  |
| --- | --- |

## Anticipation of automation effects

| Regarding mental demand I experience that automation of documentation have | Decreased demand | ________________________________________ | Increased Demand |
| --- | --- | --- | --- |
| Regarding Physical demand - I experience that automation of documentation have | Decreased demand | ________________________________________ | Increased Demand |
| Regarding temporal demands - I experience that automation of documentation have | Decreased demand | ________________________________________ | Increased Demand |
| Regarding outcome of documentation I experience that automation of documentation have | Decreased demand | ________________________________________ | Increased Demand |
| Regarding Frustration - I experience that automation of documentation have | Decreased frustration | ________________________________________ | Increased Frustration |
| Overall, I believe automation of documentation have | Decreased demand | ________________________________________ | Increased Demand |

## Open ended question

| Any other thought you want to share on automatic transfer of vital signs from the measurement device to the EHR? |  |
| --- | --- |
